# Long-term outcomes of traumatic hip dislocation: a minimum 10-year follow-up study in 18 patients

**DOI:** 10.1007/s00402-025-06048-8

**Published:** 2025-08-28

**Authors:** Stephan Regenbogen, Paul A. Grützner, Markus Beck, Philipp Blum, Ulrich Stöckle, Philipp Osten, Sven Märdian, Vera Jaecker

**Affiliations:** 1 Department for Orthopaedics and Trauma Surgery at Heidelberg University, BG Klinik Ludwigshafen, Ludwigshafen, Germany; 2 Department of Traumatology and General Surgery, BG Unfallklinik Murnau, Murnau, Germany; 3 Center for Musculoskeletal Surgery, Charitè–University Medicine Berlin, Berlin, Germany; 4 Department of Trauma and Orthopedic Surgery, Cologne Merheim Medical Center, Witten/Herdecke University, Witten, Germany; 5 Department of Trauma, Hand and Reconstructive Surgery, Rostock University Medical Center, Rostock, Germany

**Keywords:** Traumatic hip dislocation, Acetabular fractures, Pipkin fractures, Long-term outcomes, PROMs

## Abstract

**Background:**

Traumatic hip dislocations are rare but serious injuries, potentially affecting patients’ quality of life and mobility. Given the limited understanding of prognostic factors, this study aimed to identify predictors of long-term clinical outcomes.

**Materials and methods:**

Injury characteristics and computed tomography (CT) of patients following traumatic hip dislocation from two level I trauma centers from 2009 to 2015 were analyzed. At follow-up, patients were evaluated for avascular necrosis (AVN), post-traumatic osteoarthritis (PTOA), secondary surgery, complications, and return to sports. Patient-reported outcome measures (PROMs), including Tegner Activity Scale (TAS) and modified Harris Hip Score (mHHS), were evaluated.

**Results:**

38 patients with traumatic hip dislocation were finally included. Concomitant posterior acetabular wall fractures and femoral head fractures (Pipkin type I to IV) were observed in 34 cases (87%). 18 patients (mean age 38.3 ± 17.2 years) completed the follow-up (mean follow-up 12.25 ± 1.03 years). 6 patients (33%) developed PTOA, 2 patients (11%) AVN, and 3 patients (17%) required total hip arthroplasty. Decreased TAS was associated with concomitant fractures (*p* = 0.02). 10 patients (56%) did not return to their pre-injury sports level and 7 patients (39%) reported sexual dysfunction. PROMs and return to sports were significantly worse in patients with PTOA or residual sciatic nerve injury (*p* < 0.05).

**Conclusions:**

Patients after traumatic hip dislocation are at high risk for PTOA or AVN, especially with concomitant acetabular or femoral head fractures, resulting in significant long-term limitations in daily activities, sports, and sexual function. Recognition of concomitant fractures is a critical prognostic factor in assessing long-term outcomes.

## Introduction

Traumatic hip dislocations (THDs) are severe traumatological emergencies with the potential for profound impact on patients’ quality of life [1,2]. These injuries commonly result from high-energy trauma such as traffic accidents, falls from height, or high-impact sports, and often lead to significant long-term morbidity [3–5]. Among the most serious complications are avascular necrosis (AVN) of the femoral head and post-traumatic osteoarthritis (PTOA), which can manifest years after the initial injury and result in chronic pain, reduced mobility, diminished quality of life and low rates of return to work [1,6–10].

While early intervention, such as timely hip reduction, is widely regarded as a critical factor in mitigating some complications [1,11,12], the evidence base surrounding other prognostic factors including patient demographics, trauma mechanisms, and associated injuries, is notably limited. Moreover, most existing studies have been constrained by relatively short follow-up durations, heterogeneous cohorts, and an overreliance on radiographic imaging in the past [1,13,14]. These limitations have hindered a comprehensive understanding of the long-term trajectory of THD and its sequelae.

Since post-traumatic arthrosis often emerges as a late consequence, many existing studies fail to capture the delayed onset and evolution of this condition. Therefore, this study addresses the pressing need for long-term data by examining outcomes over a minimum follow-up period of 10 years, focusing on the development of late complications such as PTOA and AVN and prognostic factors. By incorporating patient-reported outcomes and functional assessments alongside epidemiological data, this study aims to identify critical prognostic indicators, such as the timing of reduction and specific injury patterns. The goal is to provide a robust framework for understanding the impact on functional recovery, return to sports, and overall quality of life. This long-term perspective is essential for guiding therapeutic strategies and improving outcomes for patients who have sustained THD.

## Methods

### Patients and study design

After receiving institutional review board approval, the records of all consecutive patients treated for traumatic dislocation of the native hip at two Level I Trauma Centers between January 1, 2009, and January 1, 2015, were reviewed.

### Inclusion and exclusion criteria

**Fig. 1:**
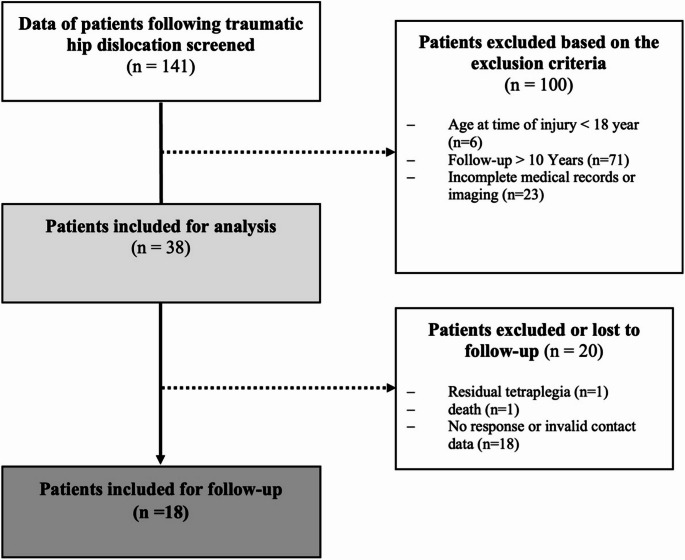
Flow chart illustrating the exclusion criteria

Patients were eligible for inclusion if they were 18 years of age or older, had closed growth plates at the time of injury, and had sustained a posterior traumatic hip dislocation with or without associated posterior acetabular wall or femoral head fracture. Other exclusion criteria included incomplete or missing pre- and post-reduction computed tomography (CT) scans. A flowchart illustrates and summarizes the exclusion process during the follow-up period **(**Fig. 1**)**.

### Data collection

Demographic data such as age, sex, and body mass index (BMI) were recorded, as well as details of the trauma mechanism, concomitant injuries, time to reduction, treatment approaches, and complications. CT scans were analyzed to determine the type of dislocation and the presence of associated femoral or acetabular fractures. Femoral head fractures were classified according to the Pipkin classification system [15].

### Follow-up and patient-reported outcome measures (PROMs)

Eligible living patients who met the inclusion criteria were contacted by the responsible orthopedic surgeon at the participating Trauma Centers. After obtaining written informed consent, patients were enrolled for follow-up. Cognitive disorders and paraplegia were exclusion criteria for follow-up. In total, 38 patients were included at baseline: 26 from hospital 1 and 12 from hospital 2. At the time of follow-up, 18 patients were available for follow-up: 14 from hospital 1 and 4 from hospital 2. The post-injury history was documented, including redislocation, hip instability, development of osteonecrosis or PTOA, additional surgical procedures, residual nerve injuries, and sexual dysfunction (erectile dysfunction in men or loss of libido in women) were assessed through a self-reported questionnaire. Return to sports and work was also assessed, noting the level of sports participation before and after the injury and whether the patient was able to return to their previous level of activity. Activity levels were quantified using the Tegner Activity Scale (TAS) [16], which rates activity on a scale of 0 to 10, with 0 representing maximum disability and 10 reflecting participation in elite national or international sports. Activities of daily living, functional outcomes, and pain levels were evaluated using the validated modified Harris Hip Score (mHHS) [17]. This score was categorized as follows: Poor < 70, Fair 70–79, Good 80–89, Excellent: ≥90.

### Statistical analysis

Descriptive statistics were used to summarize the data, including means, frequency counts, percentages, and ranges, as appropriate for continuous and categorical variables. Results were reported as mean values, standard deviations, and percentages. Φ-K multivariable correlation analysis was conducted using Python (version 3.8.16) with the Jupyter Notebook software for statistical computing to identify linear and nonlinear relationships within the data [18]. Prior to analysis, data were tested for normality using the Shapiro-Wilk test and for homoscedasticity using the Levene test. Based on these results, appropriate statistical tests were applied. Parametric tests were performed when the assumptions of normality and homoscedasticity were met. Nonparametric tests, including the Mann-Whitney U test, chi-square test, or Kruskal-Wallis test, were used when these assumptions were not satisfied. We performed a minimum detectable effect calculation for the measured variables in order to obtain an estimate of the effect size given by the small number of cases. A significance level of *p* < 0.05 was considered statistically significant.

## Results

### Demographics

Finally, 18 (44%) patients (14 male and 4 female) with a mean age of 38.33 ± 15.79 years were included in the follow-up. Most patients sustained a polytrauma in 9 (50%), followed by multiple injuries in 6 (33%) and mono hip injury in 3 (17%) patients. Concomitant posterior acetabular wall fractures and femoral head fractures (Pipkin type I to IV) were observed in the majority of cases (15 patients, 83%). Isolated THD was found in 3 cases (17%). Posterior dislocation was observed in 17 patients (94%), whereas anterior dislocation occurred in 1 patient (6%). Conservative treatment with closed reduction of the hip was performed in 5 (28%) patients and operative treatment, including open reduction and internal fixation of the femoral head or posterior acetabular wall fracture, was performed in 13 (68%) patients. Hip re-duction was performed within 6 h after the injury in 14 patients (78%) and > 6 h in 4 (22%) patients. Mean duration of the hospital stay was 18.33 ± 12.53 days. The overall demographics of the follow-up cohort are shown in Table 1.

**Table 1:** Demographics and injury characteristics of all patients following traumatic hip dislocation at time of follow-up and separated by hospitals

	Patients at follow-up *N* = 18	Patients at follow-up hospital 1 *N* = 14	Patients at follow-up hospital 2 *N* = 4
Age in years, mean ± SD	38.33 ± 15.79	42.07 ± 17.23	25.25 ± 8.64
BMI kg/m^2^, mean ± SD	24.22 ± 4.95	25.2 ± 5.23	21.53 ± 2.91
*Sex*			
Men	14 (78)	11	3
Women	4 (22)	3	1
*Side*			
Right	8 (44)	6	2
Left	10 (56)	8	2
*Time of hip reduction*			
Within 6 h	14 (78)	11	3
> 6 h	4 (22)	3	1
*Fracture classification*			
Isolated hip dislocation	3 (17)	2	1
Posterior acetabular wall fracture	7 (39)	7	0
*Pipkin fracture*			
Type I	4 (22)	3	1
Type II	1 (6)	0	1
Type IV	3 (16)	2	1
*Injury mechanism*			
Motorcycle/scooter	9 (50)	8	1
Fall/jump from height > 2 m	4 (22)	3	1
Car driver	3 (17)	1	2
Skiing	1 (6)	1	0
Fall/jump from height < 2 m	1 (6)	1	0
*Injury severity*			
Non-isolated injury	15 (83)	10	5
Isolated hip injury	3 (17)	2	1

The values are given as numbers with % in parentheses or mean values ± sd (standard deviation)

### Follow-up and Patient-Reported outcome measures (PROMs)

The mean follow-up was 12.25 ± 1.03 years. During follow-up, 1 patient (6%) reported residual subjective hip instability. 2 patients (11%) developed AVN, and 6 patients (33%) developed PTOA. Total hip arthroplasty (THA) was the most common revision surgery, performed in 3 patients (17%). Pipkin type IV and acetabular fractures were strongly associated with high rates of AVN and PTOA (*p* = 0.01). 6 patients sustained primary post-traumatic sciatic nerve injury, and 4 patients (67%) had persistent sciatic nerve palsy at follow-up. Overall, 4 patients (22%) were unable to return to their previous work. Persistent sciatic nerve palsy was associated with lower PROMs and failure to return to work (*p* = 0.04).

**Fig. 2:**
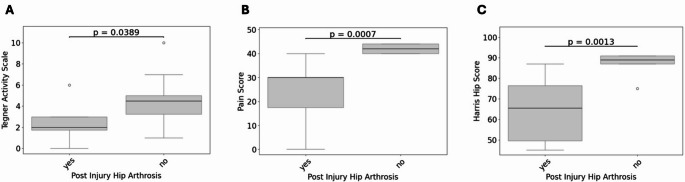
Impact of post-traumatic osteoarthritis (PTOA) on patient-reported outcome measures. Tegner Activity Scale (**A**), Pain Level (**B**), and Harris Hip Score (**C**) were significantly higher in patients with PTOA.

The median TAS decreased from 5.17 ± 2.22 before injury to 3.67 ± 2.42 at follow-up. With respect to the mHHS, nearly 60% of patients had good to excellent outcomes and approximately 28% had poor outcomes. Lower TAS was significantly associated with concomitant Pipkin type IV and posterior acetabular wall fractures (*p* = 0.02). Furthermore, lower mHHS tended to be more common in concomitant Pipkin type IV and posterior acetabular wall fractures (*p* = 0.45). 56% of patients reported limitations in returning to their pre-injury sports level, and 45% reported moderate to significant sports limitations. Post-injury sports limitations were associated with PTOA (*p* = 0.004) and lower Delta TAS (*p* = 0.01), lower mHHS (*p* = 0.02) and longer hospital stay (*p* = 0.02). In addition, decreased TAS (*p* = 0.04), mHHS (*p* = 0.001) and significantly higher levels of pain (*p* < 0.001) were significantly associated with the development of PTOA (Fig. 2A–C).

**Fig. 3:**
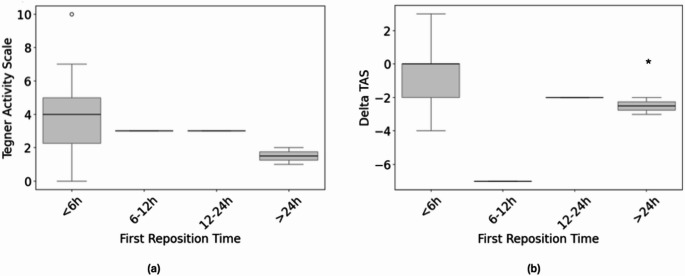
Association between timing of hip reduction and activity level before injury and at the time of follow-up. The Tegner Activity Scale (TAS) at follow-up was lower in patients with a reduction time > 6 h (**a**), and the Delta TAS from pre-injury level to follow-up was significantly greater in patients with a reduction time > 6 h after injury (**b**; *p* = 0.04)

The timing of hip reduction (within 6 h vs. >6 h) was not associated with differences in mHHS (*p* > 0.05). However, the TAS at follow-up was lower in patients with a reduction time of > 6 h (2.35 ± 0.96) compared to hip reduction within 6 h (4.07 ± 2.59), and Delta TAS was significantly higher in patients with reduction time > 6 h post injury (*p* = 0.04) (Fig. 3a, b).

Sexual dysfunction was reported by 7 patients (39%), including 5 male patients with erectile dysfunction and 2 female patients with a loss of libido. Overall clinical outcomes, including PROMs and complications, were independent of age, sex, and severity of concomitant injuries (*p* > 0.05). Overall outcome of patients at time of follow-up is presented in Table 2.

**Table 2:** Clinical outcomes and patient-reported outcome measures at follow-up and separated by hospitals

	Patients at follow-up (*n* = 18)	Patients at follow-up hospital 1 *N* = 14	Patients at follow-up hospital 2 *N* = 4
Mean follow-up	12.25 ± 1.03	12.06 ± 1.00	12.75 ± 0.75
*Return to previous work*			
Yes	14 (78)	11	3
No	4 (22)	3	1
*Revision surgery*			
No	14 (78)	11	3
Yes	4 (22)	3	1
Total hip arthroplasty	3 (17)	3	0
Hip arthroscopy	1 (5)	0	1
*Residual sciatic nerve palsy*			
Yes	6 (33)	5	1
No	2 (11)	1	1
*Tegner activity scale (TAS)*			
Pre-injury	5.17 ± 2.22	4.50 ± 1.34	7.50 ± 3.31
Time of follow-up	3.67 ± 2.42	3.00 ± 1.88	6.00 ± 2.94
*Limitations in sports activity*			
No	8 (44)	7	1
Low	2 (11)	0	2
Moderate	3 (17)	2	1
Severe	5 (28)	5	0
*Modified Harris hip score (mHHS)*			
< 70 (poor result)	5 (28)	4	1
70–79 (fair result	2 (11)	2	0
80–89 (good result)	6 (33)	3	3
> 90 (excellent result)	5 (28)	5	0
*Sexual dysfunctio*n			
Yes	7 (39)	6	1
No	11 (61)	8	3

The values are given as numbers with % in parentheses

## Discussion

The findings of this study provide valuable insight into the long-term complications associated with traumatic hip dislocation after a minimum of ten years of follow-up. The most notable findings were that 44% of the patients developed PTOA or AVN over time, often requiring THA. Furthermore, associated Pipkin type IV and acetabular fractures had significantly worse outcomes in TAS, mHHS and overall sports limitations. In addition, the timing of hip reduction was not associated with the development of PTOA or AVN, but late reduction was correlated with a decreased TAS.

THDs are commonly associated with unsatisfactory clinical outcomes and overall limitations in activities of daily living [1,2,4,13]. Among the most severe complications is PTOA, which may develop years after the initial trauma and lead to chronic pain, reduced mobility, and decreased quality of life [1,6–8]. However, the broader landscape of prognostic factors, including patient demographics, mechanism of injury, and concomitant injuries, remains insufficiently understood. In addition, many previous studies suffer from limitations such as small patient cohorts, short follow-up periods, heterogeneous patient populations, and an overreliance on radiographic assessments [13,14]. These limitations have hindered a comprehensive evaluation of the long-term course of THD and its complications. To provide a comprehensive overview of the existing evidence, we conducted a structured literature review spanning the years 1995 to 2025. The relevant studies were identified and their key findings summarized in Table 3.

**Table 3:** Summary of the relevant previously published studies on clinical outcomes after traumatic hip dislocation, including author, time to follow-up, complications, outcome, and conclusion, as reported in the medical literature

Authors and year	Patients (*n*)	Time to follow-up (mean)	PTOA, AVN, HO, nerve injury, residual pain	Outcome	Conclusion
Dreinhöfer et al. (1994)	42	8.0 years	PTOA 16.6%, AVN 4.8%, HO 9.5%	Fair and poor results in 45% (clinical) and 26% (radiographically) according to Thomson and Epstein	Posterior dislocations and severe concomitant injuries were associated with a higher rate of fair or poor outcomes, underscoring the importance of injury severity in prognosis.
Sahin et al. (2003)	62	9.6 years	PTOA 16.1%, AVN 8.1%, HO 6.5%	Fair and poor results in 29% according to Merle d’Aubigne and Postel	Early, stable, and accurate reduction, leads to good outcomes. Concentric reduction must be confirmed radiological.
Dwyer et al. (2006)	35	4.6 years	PTOA 25.7%, AVN 28.5%, HO 14.2%, nerve injury 11.4%	Not collected	Type IV posterior hip dislocations are associated with the highest complication rate, including avascular necrosis, osteoarthritis, sciatic nerve injury, and heterotopic ossification, even with timely reduction.
Al-Bahlool et al. (2009)	58	4.9 years	PTOA 8.6%, AVN 3.4%, residual pain 13.8%,	Not specified according to Merle d’Aubigne and Postel	Early reduction combined with immobilization for at least four weeks leads to favourable outcomes in traumatic hip dislocation, while shorter immobilization periods are associated with poorer results.
Pascarella et al. (2019)	33	3.0 years	PTOA 30.3%, AVN 3%, HO 3%, nerve injury 3%	Fair and poor results in 45% (clinical) and 48% (radiographically) according to Thomson and Epstein	Post-traumatic osteoarthritis is the most frequent complication of hip dislocations, followed by avascular necrosis and heterotopic ossification. Prognosis is primarily determined by the severity of the initial trauma rather than the surgical approach.
Milenkovic et al. (2022)	44	5.4 years	AVN 20.4%	Moderate and poor results in 30% according to Harris hip score (with acetabular fracture)	Delayed hip reduction significantly increases the risk of avascular necrosis, especially in posterior fracture dislocation. Timely reduction, along with anatomical fracture fixation and early stable osteosynthesis, is critical for optimal outcomes.
Jaecker et al. (2024)	112	6.0 years	PTOA 32%, AVN 13%, nerve injury 27,5%	Fair and poor results in 42% according to modified Harris hip score	Traumatic hip dislocations, especially those involving Pipkin Type IV or acetabular rim fractures, are linked to long-term functional limitations and carry a higher risk of requiring total hip arthroplasty due to osteonecrosis or post-traumatic osteoarthritis, as well as increased risk of sciatic nerve injury.

*AVN* Avascular necrosis, *PTOA* post-traumatic osteoarthritis, *ho* heterotopic ossification

The current study demonstrated a high long-term incidence of post-traumatic osteoarthritis and avascular necrosis in nearly half of patients following THD. Previous studies have reported high rates of AVN after THD [1,7,19] Thereby, the severity of the initial injury correlates with a higher risk of developing AVN and PTOA [20]. Rodriguez et al. observed PTOA in up to 88% of patients with THD and associated acetabular fractures [21]. Moreta et al. demonstrated excellent outcomes in patients with simple dislocations without radiographic evidence of osteoarthritis, whereas complex dislocations were associated with significantly worse functional and radiological outcomes [22]. In our cohort, isolated traumatic hip dislocations without associated fractures were relatively rare (17%) and showed no association with PTOA or AVN. In contrast, femoral head fractures were identified in 40% of cases, with Pipkin type IV fractures being the most common. These injuries were strongly associated high rates of AVN and PTOA and the worst functional outcomes.

These findings highlight the long-term challenges patients face after traumatic hip dislocation and emphasize the critical importance of comprehensive imaging to accurately identify associated fractures that may be missed on conventional radiographs. A long-term, holistic approach is essential to understanding how complications such as AVN and PTOA impact functional status, return to sports, and overall quality of life. This understanding is fundamental to developing evidence-based treatment strategies and improving long-term patient outcomes associated with traumatic hip injuries.

Only adult patients were included in this study and all patients experienced a high-energy trauma. Although low-energy mechanisms are frequently described in the literature, these often affect children or adolescents and are associated with predisposing factors like increased acetabular retroversion, hip dysplasia or femoroacetabular impingement [23–27]. A recent study analyzed 66 children and adolescents who sustained hip dislocation and in patients ≤ 10 years old, 66.7% had a low-energy trauma and in patients > 11 years old a high-energy trauma was observed only in 61.9% of the cases [28]. This highlights the likelihood of other factors contributing to hip dislocation in younger patients. To eliminate potential outcome bias from growth-related factors, patients under 18 years of age were excluded from this study.

Activities of daily living and sports participation showed notable deterioration during the follow-up. Limitation in sports activities after injury was associated with PTOA and lower Delta TAS, lower mHHS and longer hospital stay. Furthermore, lower TAS and mHHS were significantly associated with PTOA. The decrease in TAS and the fact that almost one third of patients had poor results in mHHS indicate a notable deterioration in patients’ ability to participate in daily activities and sports, which can have a significant impact on their quality of life. In addition, the reported sexual dysfunction in 39% of patients, including erectile dysfunction in men and loss of libido in women, points to the multifaceted consequences of hip injuries that extend beyond physical limitations.

The majority of patients underwent hip reduction within 6 h post-injury. This timely intervention has been associated with in minimizing certain complications [1,11,29]. We found that the Delta TAS was significantly higher in patients with later reduction time after injury. However, the timing of hip reduction was not associated with differences in mHHS or in the development of PTOA or AVN. While previous studies have suggested that early reduction reduces the risk of AVN, especially when completed within 6 h [11,12] others found that the interval to reduction might be less important than previously described [7,30,31]. Therefore, the safe interval between injury and hip reduction remains controversial, especially since most studies have not considered other factors that may influence the risk of AVN or PTOA. Furthermore, due to the relatively small number of patients with late reduction and the numerous other confounding factors in the present study, it was difficult to determine whether late reduction was an independent risk factor for AVN or PTOA. Ensuring the earliest possible hip reduction is critical to reducing the duration of compromised blood supply to the femoral head, but other factors may play a more critical role in recovery and long-term outcome.

This study has strengths and limitations. The major strength is that it is the first study evaluating outcomes following traumatic hip dislocation with a minimum of 10 years of follow-up. Furthermore, the classification of dislocation types and associated avulsion fractures was conducted with enhanced accuracy using CT scans in all cases, which provides superior diagnostic accuracy compared with conventional radiographic imaging. Nonetheless, the study is not without limitations. One significant limitation of the study is that 56% of the patients were lost to follow-up. Additionally, only 18 patients completed the follow-up. Based on the observed effect sizes, the limited sample size may have compromised the ability to detect clinically meaningful differences with adequate statistical power between subgroups. However, due to the scarcity of long-term follow-up studies on traumatic hip dislocation, we focused on including only those patients who had a minimum follow-up of 10 years. Furthermore, different treatment algorithms among the centers may have affected the results. In addition, the heterogeneity in injury types, ranging from isolated hip dislocations to complex Pipkin fractures, as well as the diversity in treatment strategies (operative vs. non-operative) introduce variability that may influence outcomes independently.

## Conclusions

In conclusion, traumatic hip dislocations often result in long-term limitations in daily activities, work, sports, and sexual function. Patients, particularly those with posterior wall or femoral head fractures, are at high risk for PTOA or AVN and demonstrate reduced PROMs and sports participation. Recognizing concomitant fractures is therefore a critical prognostic factor in assessing the long-term outcome of traumatic hip dislocation.

## Data Availability

No datasets were generated or analysed during the current study.
